# The prognostic significance of preoperative serum albumin in urothelial carcinoma: a systematic review and meta-analysis

**DOI:** 10.1042/BSR20180214

**Published:** 2018-07-06

**Authors:** Jing Liu, Fang Wang, Shaohong Li, Wenhui Huang, Yanjuan Jia, Chaojun Wei

**Affiliations:** 1 Department of Clinical Laboratory Center, Gansu Provincial Hospital, 204 Donggang West Road, Lanzhou 730030, China; 2 Department of Nephrology, Gansu Provincial Hospital, 204 Donggang West Road, Lanzhou 730030, China; 3 The Institute of Clinical Research and Translation Medicine, Gansu Provincial Hospital, 204 Donggang West Road, Lanzhou 730030, China

**Keywords:** Albumin, meta-analysis, prognosis, urothelial carcinoma

## Abstract

Preoperative serum albumin has been considered to be closely correlated with the prognosis of various cancers, including urothelial carcinoma (UC). However, to date, this conclusion remains controversial. The aim of this meta-analysis is to investigate the prognostic significance of preoperative serum albumin in UC. A literature search was performed in PubMed, Web of Science, Embase, and Cochrane Library up to 4 July 2017. Herein, a total of 15506 patients from 23 studies were enrolled in our meta-analysis. Decreased preoperative serum albumin level predicted poor overall survival (OS) (HR = 1.88, 95% CI: 1.44–2.45, *P*<0.0001), cancer-specific survival (CSS) (HR = 2.03, 95% CI: 1.42–2.90, *P*=0.0001), recurrence-free survival (HR = 1.85, 95% CI: 1.15–2.97, *P*=0.01), 30-day complications (30dCs) after surgery (odds ratio (OR) = 1.93, 95% CI: 1.16–3.20, *P*=0.01), and 90-day mortality after surgery (OR = 4.24, 95% CI: 2.20–8.16, *P*<0.001). The subgroup analyses indicated that low preoperative serum albumin level is still positively associated with a worse prognosis of UC based on ethnicity, cut-off value, tumor type, analyses type, and sample size. Our meta-analysis indicated that reduced preoperative serum albumin level was a predictor of poor prognosis of UC.

## Introduction

Urothelial carcinoma (UC), the major histologic type of bladder cancer (BC), is one of the most common and fatal types of genitourinary tract malignancies [1], while upper tract UC (UTUC) makes up only 5–10% of UC with a poor prognosis [2–4]. The standard treatments for UC are radical resection including radical cystectomy (RC) with pelvic lymph node (LN) dissection for BC and radical nephroureterectomy (RNU) coupled with excision of a bladder cuff for UTUC [2,5]. However, radical surgery correlates with a high incidence of early postoperative complications [6,7] and mortality [6,8]. Even worse, tumor recurrence occurs in more than 20% of patients within 10 years of the operation [7,9,10]. Therefore, it is imperative to establish an effective prognostic model to stratify patients and then make a plan for an optimal preoperative management. Presently, postoperative TNM stage and grade are the factors that are most widely used to stratify UC patients, but their accuracy may be unsatisfactory. Additional predictive factors should be explored to solve the intractable clinical problem.

Serum albumin, the main serum protein [11], can be tested to estimate visceral protein function. The normal level of serum albumin for an adult varies from 3.5 to 5.0 g/dl, and the definition of hypoalbuminemia is <3.5 g/dl [12,13]. It has been demonstrated that albumin synthesis can be inhibited by malnutrition and inflammation during the later stages of disease [14,15]. Moreover, inflammation is a key step in the development and progression of cancers [16,17]. In recent years, many studies have indicated that preoperative serum albumin level can serve as an indicator of inflammation [18,19] and is closely related to the prognosis of various types of cancers [20–22]. In particular, the link between the preoperative serum albumin level and the prognosis of UC patients has been widely investigated in many studies [23–27]. Nevertheless, the prognostic significance of preoperative serum albumin level in UC patient remains controversial. For instance, some studies reported that preoperative serum albumin can act as a predictor of the prognosis of UC patients [24,28,29], but the conclusion was different in the other studies [27,30,31]. Hence, we performed a systematic review and meta-analysis to assess the prognostic significance of preoperative serum albumin in UC patients.

## Materials and methods

### Publication search strategy

A comprehensive literature search was conducted in PubMed, Embase, Web of Science, and Cochrane library up to 4 July 2017. The following terms were used to perform the search: ‘urothelial carcinoma or urothelial cancer or bladder cancer or upper tract urothelial carcinoma or radical cystectomy or radical nephroureterectomy’ and ‘albumin or serum albumin or hypoalbuminemia’ and ‘prognosis or survival or outcome or prognostic’. The search was limited to articles published in English.

### Inclusion criteria

The enrolled studies were required to meet the following criteria: (i) the diagnosis of UC patients was histopathologically validated; (ii) preoperative serum albumin was measured and the correlation with prognosis of UC patients was analyzed; and (iii) the full text of publications should be available in order to access the data. The exclusion criteria were as follows: (i) reviews, case reports, animal research, letters, and meeting abstracts; and (ii) the same institution or authors between articles, as this may result in duplicate data.

### Data extraction

The eligible relevant data were extracted from the included articles by two independent investigators. Any disagreements encountered during data extraction were resolved through a consensus. The extracted data included the author, year, number of cases, sex, the use of neoadjuvant chemotherapy, follow-up duration, cut-off value, and the end points. We were interested in the overall survival (OS), cancer-specific survival (CSS), recurrence-free survival (RFS), 30-day complication (30dC), and 90-day mortality (90dM).

If data from multivariable and univariate analyses were both available in the publications, the former was chosen. Survival data were extracted by applying Engauge Digitizer (version 4.1) if studies only included Kaplan–Meier curves. The HRs and their 95% CIs for prognosis were estimated according to the Tierney et al. [32] methods.

### Quality assessment

The quality of included articles was evaluated independently by two investigators using the Newcastle–Ottawa Scale (NOS), in which the scores ranged from 0 to 9 [33]. In present meta-analysis, a study was regarded as high quality if it obtained 6 or more points.

### Statistical analysis

This meta-analysis was carried out using Review sManager 5.0 (Cochrane Collaboration, Oxford, U.K.) and Stata SE12.0 (StataCorp, College Station, TX). The association between preoperative serum albumin and survival outcomes of UC patients was described by HRs with 95% CIs. In addition, odds ratios (ORs) with 95% CIs were used for the description of the relationship between early postoperative outcomes and preoperative serum albumin. Chi-square-based *Q* and *I^2^* tests were used to estimate the heterogeneity amongst the studies. In the present study, *I^2^* > 50% and *P*<0.05 indicate that a significant statistical heterogeneity exists. A random effects model was applied to pool the data if heterogeneity was significant. On the contrary, a fixed effects model was used when there is no significant heterogeneity.

Subgroup analysis, meta-regression, and sensitivity analysis were conducted to explore the possible source of heterogeneity, while also determining whether our pooled analyses were robust. The subgroup analysis and meta-regression were performed according to tumor type, ethnicity, analysis type, cut-off value, and sample size, and the sensitivity analysis was performed by omitting a single study in each step.

### Publication bias

Begg’s and the Egger’s tests [34] were used to test the publication bias for the outcomes mentioned in the least included studies. Significant publication bias was considered to exist if the funnel plot was asymmetric and the *P*-values in Egger’s or Begg’s test are less than 0.05.

## Results

### Search results and characteristics of eligible articles

**Figure 1:**
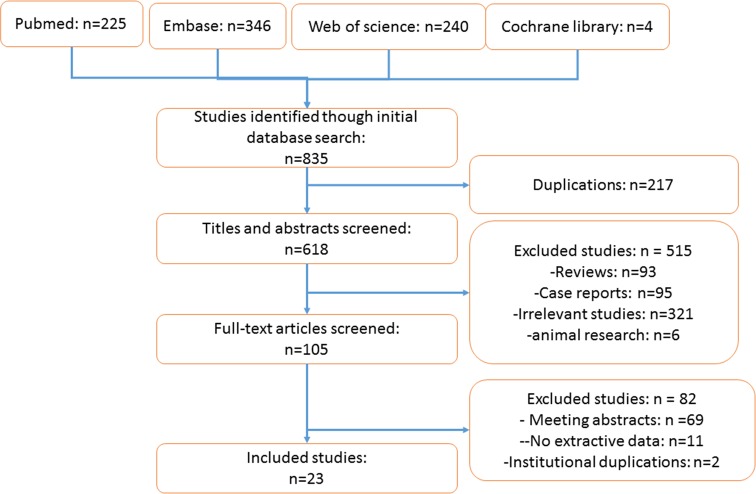
The study flow of study selection process

The publication search found 835 potentially relevant articles, but only 23 eligible studies with 15506 patients were finally included in this meta-analysis. The study selection process is shown in Figure 1.

The detailed information on the eligible articles is presented in Table 1. The main characteristics that we were interested in included country, tumor type, case number, age, sex, the use of neoadjuvant chemotherapy, follow-up duration, and cut-off value. The 23 studies used for the meta-analysis included 4 from Japan, 4 from China, 2 from Korea, 9 from the U.S.A., 1 from Britain, 1 from France, 1 from Turkey, 1 from Egypt, and 1 from Canada. Amongst the studies, 18 analyzed the prognostic value of preoperative serum albumin in participants with BC, 5 with UTUC, and 1 with both BC and UTUC. Meanwhile, the relationship between preoperative serum albumin and OS was assessed in 12 studies; CSS was analyzed in 9 studies; RFS was reported in 6 studies; 30dCs after surgery were evaluated in 7 studies; and 90dM after surgery was reported in 6 studies (Table 2). HRs were directly presented in 15 studies, and there were 2 articles only providing Kaplan–Meier curves, which could be used to estimate HRs. In addition, the quality of the included studies was assessed according to the NOS, and the scores ranged from 5 to 7, with a mean of 6.5, indicating that the included studies had moderate to high quality (Table 3).

**Table 1:** Main characteristics of the included studies

Study	Country	Tumor type	Case number (LSA/HSA)	Age (years)	Sex (M/F)	Neoadjuvant chemotherapy (LSA/HSA)	Surgical treatment	Follow-up (months)	Survival analysis	Cut-off value
Caras et al. (2017) [23]	U.S.A.	BC and UTUC	1292/4282	65	4228/1340	NA	RC or TURBT	1	NA	3.5 g/dl
Chan et al. (2013) [35]	China	BC	62/55	68 ± 10	99/18	NA	RC	31 ± 29	CSS, OCS, OS	3.9 g/l
Cho et al. (2014) [30]	Korea	UTUC	35/112	70	40/106	NA	RNU	33	RFS	3.5 g/dl
Cui et al. (2017) [24]	China	UTUC	54/36	65.66	107/62	NA	RNU	53.7	OS, CSS	4.37 g/l
Djaladat et al. (2014) [28]	U.S.A.	BC	197/1274	67	1154/317	15/92	RC	148.8	OS, RFS	3.5 g/dl
Fujita et al. (2015) [36]	Japan	UTUC	NA	70	221/85	NA	RNU	41	RFS, CSS	NA
Garg et al. (2014) [37]	U.S.A.	BC	150/947	68	831/266	19/109	RC	25.2	NA	4.0 g/dl
Hinata et al. (2015) [31]	Japan	BC	NA	68.6	575/155	NA	RC	52	OS, RFS	3.5 g/l
Huang et al. (2017) [25]	China	UTUC	17/408	67	279/146	NA	RUN	38.5	OS, CSS	3.5 g/l
Johnson et al. (2015) [38]	U.S.A.	BC	102/587	73	530/159	NA	RC	1	NA	3.5 g/dl
Kluth et al. (2014) [39]	U.S.A.	UTUC	NA	70	175/67	NA	RNU	9	CSS	3.7 g/dl
Krane et al. (2013) [29]	U.S.A.	BC	NA	67.4	55/13	NA	RC	25	OS, CSS	3.5 g/dl
Ku et al. (2015) [40]	Korea	BC	NA	65.1	362 /57	NA	RC	37.7	OS, CSS	3.5 g/dl
Lambert et al. (2013) [41]	Britain	BC	31/156	67.4	153/34	29/6	RC	26.2	OS, CSS	3.5 g/dl
Laurent et al. (2017) [26]	France	BC	95/98	75.2	164/29	NA	RC	9.1	OS	3.5 g/dl
Lavallee et al. (2014) [42]	Canada	BC	341/1090	70	1819/484	NA	RC	NA	NA	3.7 g/dl
Liu et al. (2016) [43]	China	BC	129/167	61.71	250/45	NA	RC	72	RFS, CSS	4.0 g/dl
Morgan et al. (2011) [44]	U.S.A.	BC	30/139	78.8	122/47	NA	RC	3	NA	3.7 g/dl
Mursi et al. (2013) [45]	Egypt	BC	24/7	58.4	22/9	NA	RC	3	NA	3.5 g/dl
Nakagawa et al. (2017) [27]	Japan	BC	NA	69	248/58	NA	RC	6.8	OS	3.5 g/dl
Sharma et al. (2016) [46]	U.S.A.	BC	NA	70.1	209/65	NA	RC	NA	NA	4.0 g/dl
Sheth et al. (2016) [47]	U.S.A.	UTUC	NA	71	77/24	NA	RNU or partial ureterectomy	18.5	RFS, OS	4.0 g/dl
Taguchi et al. (2016) [48]	Japan	UC	NA	68	160/40	NA	RNU and RC	12	OS	3.5 g/dl

Abbreviations: HSA, high serum albumin; LSA, low serum albumin; NA, not available; TURBT, transurethral resection of bladder tumor.

**Table 2:** The interest outcomes extracted from included studies

Study	HR	HR	HR	OR	OR
	(95% CI) for OS	(95% CI) for CSS	(95% CI) for RFS	(95% CI) for 30dC	(95% CI) for 90dM
Caras et al. (2017)	NA	NA	NA	3.14 (2.86, 3.45) (overall morbidity)*	7.66 (5.80, 10.12) (overall morbidity)*
				1.85 (1.41, 2.44) (RC)*	1.71 (0.85, 3.45) (RC)*
				4.32 (3.47, 5.39) (TURBT)*	9.89 (6.05, 16.16)(TURBT)*
Chan et al. (2013)	NA	1.79 (0.78, 4.08)*	NA	NA	NA
Cho et al. (2014)	0.60 (0.26–1.39)	NA	2.88 (1.80–4.62)	NA	NA
Cui et al. (2017)	5.509 (2.144–14.158)	5.521 (2.074–14.697)	NA	NA	NA
Djaladat et al. (2014)	1.93 (1.43–2.63)	NA	1.68 (1.16–2.43)	1.41 (0.98–2.02)	2.42 (1.31, 4.45)*
Fujita et al. (2015)		2.63 (1.149, 6.02)	2.63 (5.882, 1.149)		
Garg et al. (2014)	NA			1.68 (1.17, 2.41)	3.03 (7.143, 1.33)
Hinata et al. (2015)	1.062 (0.643–1.703)	NA	1.077 (0.654–1.718)	NA	NA
Huang et al. (2017)	1.96 (0.96–4.01)	2.51 (1.22–5.18)	NA	NA	NA
Johnson et al. (2015)	NA	NA	NA	1.79 (1.06, 3.03)	NA
Kluth et al. (2014)	NA	1.754 (1.2987, 2.326)	NA	NA	NA
Krane et al. (2013)	4.96 (2.18–11.67)	8.10 (2.63–27.59)	NA	NA	NA
Ku et al. (2015)	1.670 (1.007–2.767)	1.794 (1.010–3.187)	NA	NA	NA
Lambert et al. (2013)	1.76 (1.03, 2.12)*	1.57 (1.24, 1.90)*	NA	3.9 (1.3–12.2)	22.96 (2.47, 213.36)*
Laurent et al. (2017)	3.06 (1.81–5.17)	NA	NA	NA	NA
Lavallee et al. (2014)	NA	NA	NA	1.16 (1.06–1.26)	NA
Liu et al. (2016)	NA	0.979 (0.880–1.089)	0.998 (0.908–1.096)	NA	NA
Morgan et al. (2011)	NA	NA	NA	NA	2.50 (1.40–4.45)
Mursi et al. (2013)	NA	NA	NA	NA	9.20 (0.69, 122.38)*
Nakagawa et al. (2017)	1.51 (0.95–2.41)	NA	NA	NA	NA
Sharma et al. (2016)	NA	NA	NA	2.27 (5.56, 0.94)	NA
Sheth et al. (2016)	3.37 (1.43–7.92)	NA	4.4 (2.04–9.30)	NA	NA
Taguchi et al. (2016)	1.345 (0.969–1.855)	NA	NA	NA	NA

Abbreviation: NA, not available; TURBT, transurethral resection of bladder tumor. *Data extracted indirectly.

**Table 3:** The NOS quality assessment of the included studies

Study ID	Selection			Comparability			Outcome		Total
	Representativeness of the exposed cohort	Selection of the non-exposed cohort	Ascertainment of exposure	Demonstration that outcome of interest was not present at the start of the study	Comparability of cohorts on the basis of the design or analysis	Assessment of outcome	Was follow-up long enough for outcomes to occur	Adequacy of follow-up of cohorts	
Caras et al. (2017)	★	★	★	★	★☆	☆	★	★	7
Chan et al. (2013)	☆	★	★	★	★☆	★	★	☆	6
Cho et al. (2014)	★	★	★	★	★☆	★	★	☆	7
Cui et al. (2017)	★	★	★	★	★☆	★	★	☆	7
Djaladat et al. (2014)	★	★	☆	★	★☆	★	★	★	7
Fujita et al. (2015)	★	★	☆	★	★☆	★	★	☆	6
Garg et al. (2014)	★	★	☆	★	★☆	★	★	★	7
Hinata et al. (2015)	★	☆	★	★	★☆	★	★	★	7
Huang et al. (2017)	☆	★	★	★	★☆	★	★	★	7
Johnson et al. (2015)	★	☆	★	★	★☆	★	★	★	7
Kluth et al. (2014)	★	★	★	★	★☆	★	★	☆	7
Krane et al. (2013)	☆	☆	★	★	★☆	★	★	★	6
Ku et al. (2015)	★	★	★	★	★☆	★	★	☆	7
Lambert et al. (2013)	☆	★	★	★	★☆	★	★	★	7
Laurent et al. (2017)	★	★	★	★	★☆	★	☆	★	7
Lavallee et al. (2014)	★	★	☆	★	★☆	★	★	★	7
Liu et al. (2016)	☆	★	★	★	★☆	★	☆	★	6
Morgan et al. (2011)	☆	★	★	★	★☆	★	★	★	7
Mursi et al. (2013)	☆	★	☆	★	★☆	★	☆	★	5
Nakagawa et al. (2017)	★	★	★	★	★☆	★	★	☆	7
Sharma et al. (2016)	☆	★	☆	★	★☆	★	★	★	6
Sheth et al. (2016)	☆	★	★	★	★☆	★	★	★	7
Taguchi et al. (2016)	★	★	☆	★	★☆	★	★	★	7

★ indicates that a score (1) was assigned; ☆ indicates a score of zero.

### Preoperative serum albumin level and the survival of UC patients

**Figure 2:**
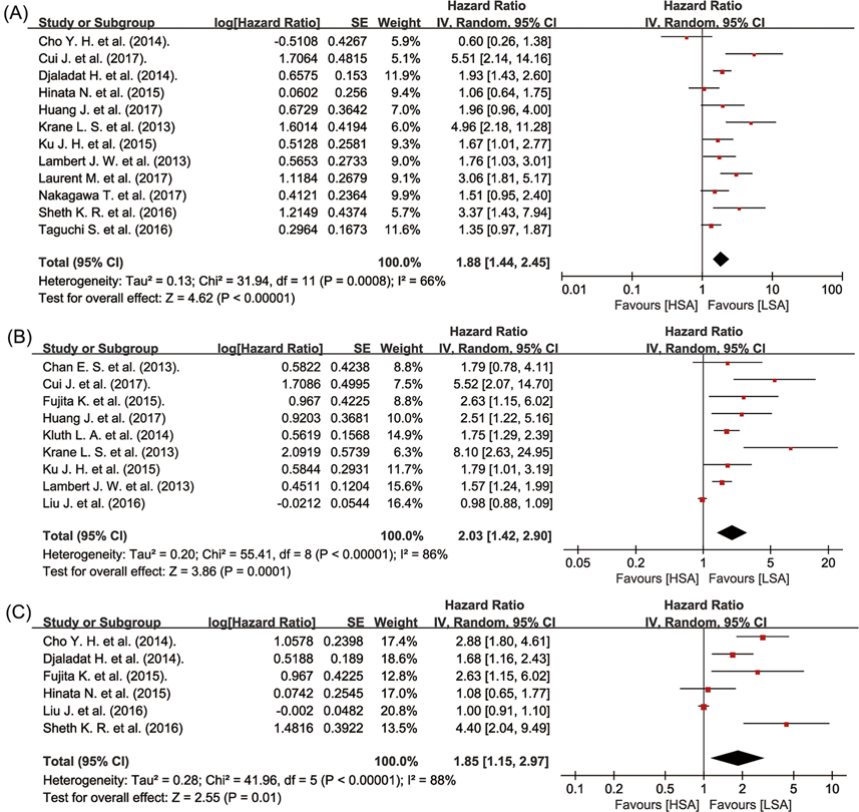
Meta-analysis of preoperative serum albumin level and OS (A), CSS (B), and RFS (C) in UC patients Abbreviations: LSA, low level of preoperative serum albumin; HSA, high level of preoperative serum albumin.

**Figure 3:**
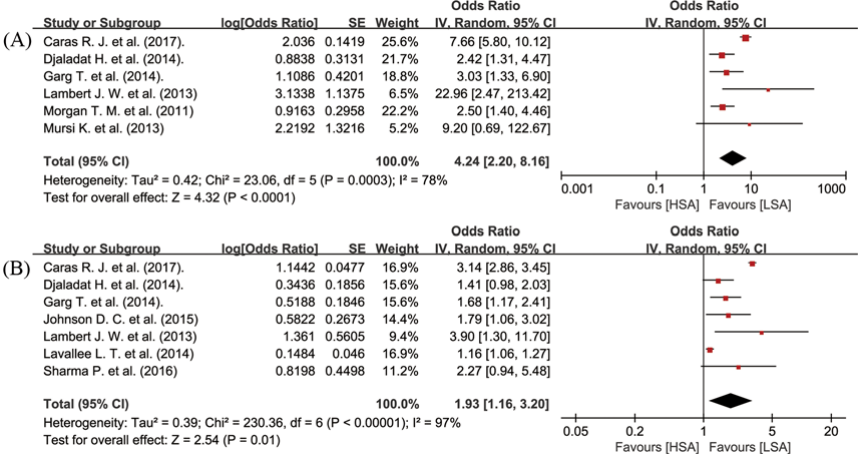
Meta-analysis of preoperative serum albumin level at 30dC (**A**) and 90dM (**B**) in UC patients

The UC patients with decreased preoperative serum albumin level suffered from significantly worse OS (random-effects model; HR = 1.88, 95% CI: 1.44–2.45, *P*<0.00001; *I^2^* = 66%, *P*=0.0008) (Figure 2A), CSS (random-effects model; HR = 2.03, 95% CI: 1.42–2.90, *P*=0.0001; *I^2^* = 86%, *P*<0.00001) (Figure 2B), and RFS (random-effects model; HR = 1.85, 95% CI: 1.15–2.97, *P*=0.01; *I^2^* = 88%, *P*<0.00001) (Figure 2C). With respect to short-term outcomes after surgery, low preoperative serum albumin level was significantly related to a lower 90dM after surgery (random-effects model; OR = 4.24, 95% CI: 2.20–8.16, *P*<0.0001; *I^2^* = 78%, *P*=0.0003) (Figure 3A), and a lower rate of 30dCs after surgery (random-effects model; OR = 1.93, 95% CI: 1.16–3.20, *P*=0.01; *I^2^* = 97%, *PP*<0.00001) (Figure 3B).

### The analysis of potential sources of heterogeneity

**Figure 4:**
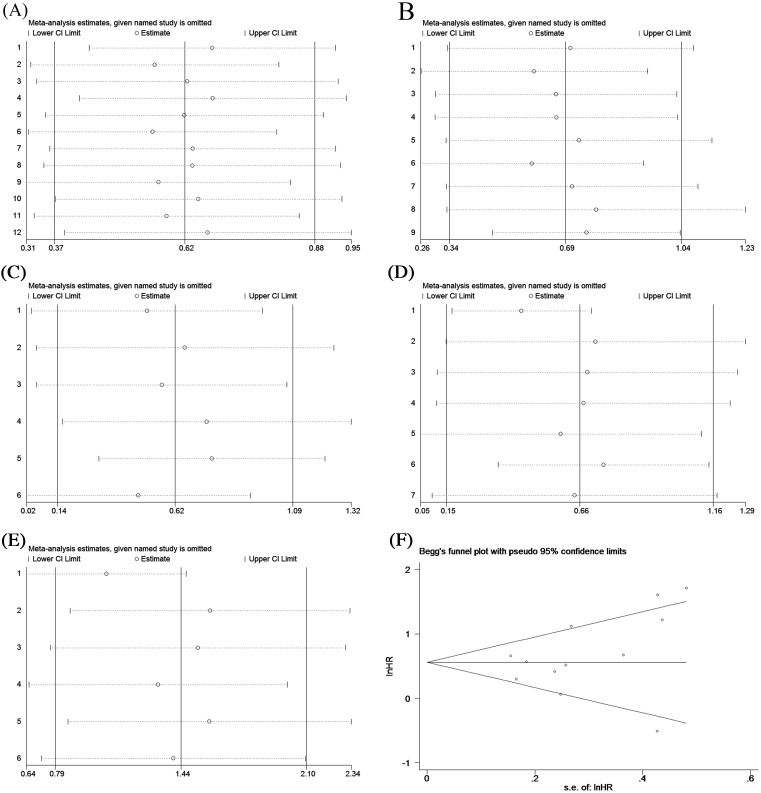
Sensitivity analyses for OS (**A**), CSS (**B**), RFS (**C**), 30dC (**D**), 90dM (**E**), and the Begg’s and Egger’s test results for UC patients’ OS (**F**)

To explore the potential sources of heterogeneity amongst the studies, the subgroup analysis, sensitivity analysis, and meta-regression analysis were performed in our meta-analysis. The subgroup analyses were conducted to evaluate the prognostic values of preoperative serum albumin according to ethnicity, cut-off value, tumor type, analysis type, and sample size. Our results showed that the significant heterogeneity still existed in all the subgroups, indicating that these factors might not be the sources of heterogeneity in our meta-analysis. In addition, except for RFS, the pooled results of the other outcomes in subgroup analyses did not fluctuate significantly, demonstrating the robustness of our pooled analyses. The detailed results of all the subgroup analyses are presented in Table 4. The sensitivity analyses were conducted by omitting a single study step by step and the results showed that no single study exerted significant influence on the pooled results of the outcomes, also indicating that our findings were stable (Figure 4). Next, we performed the meta-regression analysis to further explore the potential sources of heterogeneity based on five covariates, including tumor type (UTUC compared with BC), ethnicity (Asian compared with non-Asian), analysis type (univariate compared with multivariate), cut-off value (3.5 g/dl compared with others), and sample size (>600 compared with ≤600). The results indicated that tumor type accounted for most heterogeneity of the pooled HR or OR of RFS (Coef. = 0.27, 95% CI: 0.18–0.79, *P*=0.02), 30dC (Coef. = 0.48, 95% CI: 0.25–0.92, *P*=0.03), and 90dM (Coef. = 0.36, 95% CI: 0.18–0.74, *P*=0.02), and explained 84.61, 69.5, and 100% between-study variance in RFS, 30dC, and 90dM, respectively. The other covariates could not clarify the heterogeneity, and the details of meta-regression were presented in Table 5.

**Table 4:** Subgroup analysis of preoperative serum albumin and the prognosis of UC patients

Variables	Outcome	Studies	Patients	HR (95% CI)	*P*-value	Model	Heterogeneity	
							*I^2^* (%)	*P*-value
**Tumor type**								
UTUC	OS	4	841	2.03 (1.34, 3.07)	<0.01	Random	78	<0.01
	CSS	6	900	1.69 (1.16, 2.46)	<0.01	Random	87	<0.01
	RFS	3	553	3.11 (2.17, 4.46)	<0.01	Fixed	0	0.59
	30dC	1	5735	3.14 (2.86, 3.45)	-	-	-	-
	90dM	1	2669	7.66 (5.80, 10.12)	-	-	-	-
BC	OS	8	3288	1.80 (1.38, 2.33)	<0.01	Random	60	0.01
	CSS	6	1328	1.69 (1.16, 2.46)	<0.01	Random	87	<0.01
	RFS	3	2210	1.19 (0.85, 1.68)	0.32	Random	72	0.03
	30dC	6	5568	1.55 (1.19, 2.02)	<0.01	Random	62	0.02
	90dM	5	5568	2.87 (1.89, 4.36)	<0.01	Fixed	14	0.33
**Ethnicity**								
Asian	OS	6	1665	1.59 (1.09, 2.32)	0.02	Random	62	0.02
	CSS	6	1731	2.03 (1.17, 3.54)	0.01	Random	82	<0.01
	RFS	4	1477	1.60 (0.90, 2.86)	0.11	Random	87	<0.01
Non-Asian	OS	6	2271	2.20 (1.50, 3.23)	<0.01	Random	67	0.01
	CSS	3	497	2.02 (1.28, 3.19)	<0.01	Random	75	0.02
	RFS	2	1286	2.56 (1.00, 6.52)	0.05	Random	80	0.03
**Analysis type**								
Univariate	OS	10	3859	1.70 (1.31, 2.20)	<0.01	Random	62	<0.01
	CSS	4	953	2.25 (1.46, 3.47)	<0.01	Fixed	43	0.15
	RFS	2	247	1.64 (0.23, 11.53)	0.62	Random	92	<0.01
	30dC	2	6939	2.35 (1.43, 3.87)	<0.01	Random	82	<0.01
	90dM	5	7140	4.64 (2.15, 10.03)	<0.01	Random	82	<0.01
Multivariate	OS	2	270	4.21 (2.23, 7.94)	<0.01	Fixed	0	0.45
	CSS	5	1275	1.81 (1.14, 2.87)	0.01	Random	88	<0.01
	RFS	4	2516	1.33 (0.91, 1.94)	0.13	Random	75	<0.01
	30dC	5	4364	1.46 (1.06, 2.00)	0.02	Random	62	0.05
	90dM	1	1097	3.03 (1.33, 6.90)	-	-	-	-
**Cut-off value**								
=3.5 g/dl	OS	10	3859	1.70 (1.31, 2.20)	<0.01	Random	62	<0.01
	CSS	5	1216	2.11 (1.40, 3.17)	<0.01	Random	55	0.06
	RFS	3	2061	1.74 (1.04, 2.91)	0.03	Random	75	0.02
	30dC	5	8726	2.09 (1.35, 3.25)	<0.01	Random	87	<0.01
	90dM	5	8068	4.92 (2.35, 10.31)	<0.01	Random	74	<0.01
Others	OS	2	270	4.21 (2.23, 7.94)	<0.01	Fixed	0	0.45
	CSS	4	1012	1.92 (1.06, 3.47)	0.03	Random	89	<0.01
	RFS	3	702	2.14 (0.77, 5.97)	0.15	Random	90	<0.01
	30dC	2	2577	1.40 (0.78, 2.52)	0.27	Random	55	0.14
	90dM	1	169	2.50 (1.40, 4.46)	-	-	-	-
**Sample size**								
*n*>600	OS	2	1915	1.48 (0.83, 2.65)	0.18	Random	75	0.05
	RFS	4	1915	2.30 (1.02, 5.18)	0.13	Random	49	0.16
	30dC	5	10842	1.73 (0.98, 3.08)	0.06	Random	98	<0.01
	90dM	3	387	4.01 (1.72, 9.32)	<0.01	Random	86	<0.01
*n*<600	OS	10	2214	2.02 (1.45, 2.81)	<0.01	Random	67	<0.01
	RFS	2	848	1.39 (0.90, 2.14)	0.05	Random	92	<0.01
	30dC	2	461	2.81 (1.41, 5.58)	<0.01	Fixed	0	0.45
	90dM	3	7850	5.68 (1.32, 24.41)	0.02	Random	53	0.12

**Table 5:** Assessment of potential sources of heterogeneity amongst studies by meta-regression

Covariates	Outcomes	*P*-value	Regression coefficient (95% CI)
**Tumor type (UTUC compared with BC)**			
	OS	0.82	0.92 (0.36–2.37)
	CSS	0.15	0.52 (0.20–1.35)
	RFS	0.02	0.27 (0.18–0.79)
	30dC	0.03	0.48 (0.25–0.92)
	90dM	0.02	0.36 (0.18–0.74)
**Ethnicity (Asian compared with non-Asian)**			
	OS	0.10	1.68 (0.90–3.14)
	CSS	0.81	1.11 (0.39–3.16)
	RFS	0.43	1.58 (0.37–6.80)
**Analysis type (univariate compared with multivariate)**			
	OS	0.74	0.88 (0.36–2.10)
	CSS	0.54	0.77 (0.29–2.04)
	RFS	0.05	0.39 (0.19–1.02)
	30dC	0.16	0.62 (0.30–1.30)
	90dM	0.66	0.66 (0.06–7.13)
**Cut-off value (3.5 g/dl compared with others)**			
	OS	0.08	2.50 (0.89–7.01)
	CSS	0.72	0.86 (0.32–2.30)
	RFS	0.78	1.18 (0.27–5.22)
	30dC	0.32	0.64 (0.21–1.92)
	90dM	0.42	0.52 (0.07–4.04)
**Sample size (>600 compared with ≤600)**			
	OS	0.46	1.38 (0.54–3.49)
	RFS	0.40	1.63 (0.38–6.97)
	30dC	0.35	1.64 (0.47–5.72)
	90dM	0.82	1.20 (0.16–9.20)

### Publication bias

As Figure 4F shows, the funnel plots for OS were almost symmetrical, and the *P*-values of Begg’s and Egger’s tests were 0.170 and 0.266, respectively, which suggested that there was no significant publication bias in our meta-analysis.

## Discussion

Numerous articles have reported that pretreatment serum albumin is correlated with the prognosis of UC patients, but the conclusions amongst studies remain inconsistent [23–31,35–48]. Therefore, we combined 23 studies with 15506 patients to perform this meta-analysis to evaluate the prognostic role of preoperative serum albumin in UC patients [23–31,35–48]. Our results demonstrated that low levels of preoperative serum albumin are significantly associated with worse OS, CSS, RFS, complications, and early mortality. In spite of significant heterogeneity, subgroup analyses conducted according to ethnicity, tumor type, analysis type, cut-off value, and sample size showed that our pooled results did not alter significantly, which indicated the robustness of the pooled results. Generally, all these findings suggested that preoperative serum albumin level played an important prognostic role in UC patients.

Although it has been demonstrated that preoperative serum albumin is closely correlated with the prognosis of UC and other cancers [20–22,24,29], the latent mechanisms remain complex and unclear. However, it is widely recognized that malnutrition and inflammation may be partly responsible for the mechanisms [49,50]. Malnutrition, partly mirrored by hypoalbuminemia, is a severe problem in cancer patients, due to a variety of mechanisms, which involve anticancer therapies and the host response to the tumor [51]. Furthermore, many unfavorable clinical consequences are related to malnutrition, including a deteriorated quality of life, an enhanced risk of chemotherapy-induced toxicity, and poor long-term survival [52]. In addition, inflammation, which is a critical step in cancer initiation and progression [16,53], can alter the levels of serum albumin [15]. Under inflammatory conditions, immune cells and tumor cells release various inflammatory mediators, including interleukin-1β, interleukin-6, and tumor necrosis factor (TNF), which can suppress albumin synthesis in liver cells [53–55]. Moreover, albumin might directly be lost from the circulatory system, since TNF can increase the permeability of capillaries [55]. In addition, previous studies have also demonstrated that serum albumin is associated with several anticancer mechanisms, including its antioxidant function [56]. Therefore, preoperative serum albumin could serve as a good predictor of the prognosis of cancers.

Serum albumin is a low cost and easily accessible predictor for UC patients, but it still does have some limitations for clinical implications. For instance, under overhydrated conditions or other disease processes, hypoalbuminemia may not indicate malnutrition [57,58], so its prognostic value in UC patients will be reduced. In addition, diet and other non-tumor-related factors can also affect the levels of serum albumin. To some degree, those factors mentioned above may explain the significant heterogeneities in our meta-analysis. Actually, to predict the prognosis of UC patients more precisely, several predictive factors involving serum albumin have been studied for clinical practice. For instance, Liu et al. [43] indicated that the albumin/globulin ratio calculated from preoperative serum albumin and globulin levels can act as an independent predictor of long-term RFS and CSS in bladder UC. Additionally, Cui et al. [24] recently reported that a predictive model based on preoperative plasma fibrinogen and serum albumin level, also known as an FA score, can be used to predict OS and CSS in UTUC. Regardless, our meta-analysis demonstrated that preoperative serum albumin plays a significant prognostic role in UC patients.

To the best of our knowledge, the present study is the first to systematically analyze the predictive value of preoperative serum albumin for prognosis of UC patients. However, the results of our meta-analysis may be challenged by some limitations. First, most of the included studies were retrospective and thus may have bias in patient selection and data analysis. Second, the cutoffs of low preoperative serum albumin were not consistent amongst the included studies. Other inconsistencies amongst the various studies included the follow-up period and the base characteristics of patients, which may cause significant heterogeneities and thus affect the robustness of the pooled analysis. In summary, heterogeneity may be the biggest limitation of our meta-analysis. Therefore, the value of preoperative serum albumin as a prognostic predictor in UC patients still requires further investigation in the future.

## Conclusion

In conclusion, this meta-analysis indicated that preoperative serum albumin is a useful predictor for the prognosis of patients with UC. The patients with decreased preoperative serum albumin have more unfavorable long-term survival and short-term outcomes. Considering the limitations in present meta-analyses, further homogeneous prospective studies are needed to confirm our findings.

## Abbreviations

BC: bladder cancer
CI: confidence interval
CSS: cancer-specific survival
HR: hazard ratio
NOS: Newcastle–Ottawa scale
OR: odds ratio
OS: overall survival
RC: radical cystectomy
RFS: recurrence-free survival
RNU: radical nephroureterectomy
TNF: tumor necrosis factor
UC: urothelial carcinoma
UTUC: upper tract UC
30dC: 30-day complication
90dM: 90-day mortality
